# Recent Advances in Agronomic and Physio-Molecular Approaches for Improving Nitrogen Use Efficiency in Crop Plants

**DOI:** 10.3389/fpls.2022.877544

**Published:** 2022-04-29

**Authors:** Talha Javed, Indu I, Rajesh Kumar Singhal, Rubab Shabbir, Adnan Noor Shah, Pawan Kumar, Dinesh Jinger, Prathibha M. Dharmappa, Munsif Ali Shad, Debanjana Saha, Hirdayesh Anuragi, Robert Adamski, Dorota Siuta

**Affiliations:** ^1^College of Agriculture, Fujian Agriculture and Forestry University, Fuzhou, China; ^2^Department of Agronomy, University of Agriculture Faisalabad, Faisalabad, Pakistan; ^3^Indian Council of Agricultural Research (ICAR)-Indian Grassland and Fodder Research Institute, Jhansi, India; ^4^Department of Plant Breeding and Genetics, Seed Science and Technology, University of Agriculture Faisalabad, Faisalabad, Pakistan; ^5^Department of Agricultural Engineering, Khwaja Fareed University of Engineering and Information Technology, Rahim Yar Khan, Pakistan; ^6^Indian Council of Agricultural Research (ICAR)-Central Institute for Arid Horticulture, Bikaner, India; ^7^Research Centre, Indian Council of Agricultural Research (ICAR)-Indian Institute of Soil and Water Conservation, Anand, India; ^8^Indian Council of Agricultural Research (ICAR)-Indian Institute of Horticultural Research, Bengaluru, India; ^9^National Key Laboratory of Crop Genetic Improvement and National Center of Plant Gene, Hubei Hongshan Laboratory, Wuhan, China; ^10^Centurion University of Technology and Management, Jatni, India; ^11^Indian Council of Agricultural Research (ICAR)- Central Agroforestry Research Institute, Jhansi, India; ^12^Faculty of Process and Environmental Engineering, Łódź University of Technology, Łódź, Poland

**Keywords:** agriculture, nitrogen use efficiency, climate change, sustainability, molecular approaches

## Abstract

The efficiency with which plants use nutrients to create biomass and/or grain is determined by the interaction of environmental and plant intrinsic factors. The major macronutrients, especially nitrogen (N), limit plant growth and development (1.5–2% of dry biomass) and have a direct impact on global food supply, fertilizer demand, and concern with environmental health. In the present time, the global consumption of N fertilizer is nearly 120 MT (million tons), and the N efficiency ranges from 25 to 50% of applied N. The dynamic range of ideal internal N concentrations is extremely large, necessitating stringent management to ensure that its requirements are met across various categories of developmental and environmental situations. Furthermore, approximately 60 percent of arable land is mineral deficient and/or mineral toxic around the world. The use of chemical fertilizers adds to the cost of production for the farmers and also increases environmental pollution. Therefore, the present study focused on the advancement in fertilizer approaches, comprising the use of biochar, zeolite, and customized nano and bio-fertilizers which had shown to be effective in improving nitrogen use efficiency (NUE) with lower soil degradation. Consequently, adopting precision farming, crop modeling, and the use of remote sensing technologies such as chlorophyll meters, leaf color charts, etc. assist in reducing the application of N fertilizer. This study also discussed the role of crucial plant attributes such as root structure architecture in improving the uptake and transport of N efficiency. The crosstalk of N with other soil nutrients plays a crucial role in nutrient homeostasis, which is also discussed thoroughly in this analysis. At the end, this review highlights the more efficient and accurate molecular strategies and techniques such as N transporters, transgenes, and omics, which are opening up intriguing possibilities for the detailed investigation of the molecular components that contribute to nitrogen utilization efficiency, thus expanding our knowledge of plant nutrition for future global food security.

## Introduction

Efforts to end hunger are as old as human civilization, and they have affected the history of humanity. The concern for global food security results primarily from saturated crop yields and also an imbalance between the supply and demand of the major food crops (wheat, rice, and maize) (DeFries et al., 2015; Wood et al., 2018). Increasing production on a sustained basis is an essential component of ensuring food security. Food production needs to increase 50% by 2030 and double by 2050 to meet projected demands (Pastor et al., 2019). Effective nutrient use especially nitrogen (N) and phosphorous (P) are important for sustained food security. A reliable supply of N and other nutrients vital for plant growth has allowed farmers to improve productivity over the past century, thus promoting economic development (Drechsel et al., 2015; McLaughlin and Kinzelbach, 2015; Reis et al., 2016).

Nitrogen (N) plays a prominent role in the plant metabolic system (Shah et al., 2021a,b). Nitrogen, is a principal component of many organic compounds (protein, nucleic acids, alkaloids enzyme) and is also associated with energy transfer molecules like adenosine diphosphate (ADP) and adenosine triphosphate (ATP) (ATP). It consists the 16% of the total protein biomass present in plants. N is also found in nucleic acids (deoxyribonucleic acid and ribonucleic acid), which play an important role in plant genetics and heredity. It is also a component of chlorophyll, which serves as a photosynthesis factory. N also has a major role in photosynthetic processes, leaf area production, leaf area duration as well as net assimilation rate that is directly related to yield enhancement (Leghari et al., 2016). More than half of the world’s population is fed by crops cultivated using synthetic nitrogen (N) fertilizers, which were made feasible by the advent of the Haber-Bosch process in the early twentieth century, which converts atmospheric nitrogen gas (N_2_) to active forms of nitrogen. Total global consumption of N fertilizer was 112.5 million tons in 2015, 118.2 million tons in 2019, and likely to reach 7.9–10.5 billion by 2050 (Zhang X. et al., 2015). The demand for huge amounts of N fertilizers is a major concern for farmers as it increases the cost of cultivation and also degrades the inherent fertility of the soil. Consequently, the use of excess N fertilizers causes the problem of N pollution, which is now considered a new threat to environmental sustainability (Kanter et al., 2020).

Nitrogen use efficiency (NUE) is defined as the yield of grain achieved per unit of N available to the crop from soil and applied fertilizer, and it can be divided into two biological components, N uptake efficiency (NUpE) which is the efficiency of absorption/uptake of supplied N, and N utilization efficiency (NUtE) which is the efficiency of assimilation and remobilization of plant N to ultimately produce grain (Han et al., 2015; Yadav et al., 2017; Congreves et al., 2021). It has been documented that the NUE value is decreased with the application of N fertilizers. For instance, in developed countries, NUE increased because of the adaption of best agronomical and fertilizer management practices, while it reduced in developing countries like India as the use of N fertilizer dramatically increased (Heffer and Prud’homme, 2016). It was reported that the world average NUE was nearly 47 and 42% in 2009 and 2010, respectively, and the major reason behind the NUE decreasing is the adoption of agronomical practices (nutrient management and varieties) associated with low NUE (Zhang X. et al., 2015). Therefore, this study primarily focused on the advanced agronomical and molecular strategies, which reduce the N fertilizer demand and enhance NUE.

Several morphological and physiological traits such as root length, lateral roots, root architecture, light capture photosynthesis, canopy height, flowering time, carbohydrate partitioning, storage, and the remobilization of N, (stem remobilization, leaf blade remobilization, and total remobilization) are linked with higher NUE in plants (Mosleth et al., 2015; Carmo-Silva et al., 2017; Guttieri et al., 2017; Tabbita et al., 2017; Hawkesford and Griffiths, 2019). Improving nitrogen use efficiency is essential for tackling the triple threat of environmental degradation, climate change, and food security. The efficiency of applied nitrogenous fertilizers is very low due to their various losses i.e., volatilization, leaching, surface runoff, and denitrification from the soil-plant system (Zhang X. et al., 2015). Nitrogen use efficiency (NUE) is a convoluted phenomenon governed by various edaphic, climatic, and management factors; it has to be managed through an amalgamated approach involving agronomical, physiological, and molecular aspects. Among these, the agronomical approach is economically feasible, ecologically viable, and sustainable. The modern fertilizers (customized fertilizer, nano fertilizers, bio-fertilizers, biochar, and zeolite) and deep fertigation helps in improving NUE in sustainable ways. Likely, the precision farming (Leaf color chart, chlorophyll meter, and remote sensing) and modern agronomical practices (Diversified crop rotation, 4R nutrient stewardship, and crop modeling) have been developed for enhancing NUE and can support good plant performance and better crop output. New technological developments need to achieve further gains by new varieties, slow-release fertilizers, nitrification and urease inhibitors, fertigation, and high-tech approaches to precision agriculture (Pires et al., 2015; Cao et al., 2017; Mylonas et al., 2020). The identification and employment of QTLs/genes/transporters which are responsible for NUE at the molecular level is now a more feasible and robust technique with the availability of efficient molecular tools (Ali et al., 2018). Previously, Zhang X. et al. (2021) employed the RNA sequencing (RNA-seq) technique to investigate the genotypic difference in response to N deficiency between two wheat NILs (1Y, high-NUE, and 1W, low-NUE). The high- and low-NUE wheat NILs showed different patterns of gene expression under N-deficient conditions, and these N-responsive genes were classified into two major classes, including “frontloaded genes” and “relatively upregulated genes.” In total, 103 and 45 genes were identified as frontloaded genes in high-NUE and low-NUE wheat, respectively. Moreover, the expression of *TaPT4*, *TaNRT2.2*, and *TaAMT1.2* was down-regulated by arbuscular mycorrhizal (AM) colonization only when roots of host plants received phosphate or nitrogen nutrient signals. However, the expression of *TaPHT1.2*, *TaNRT2.1*, and *TaNRT2.3* was down-regulated by AM colonization, regardless of whether there was nutrient transfer from AM hyphae. The expression of *TaNRT1.2* was also down-regulated by AM colonization even when there was no nutrient transfer from AM hyphae (Zhang X. et al., 2021). Nitrogen limitation adaptation (NLA) was involved in source-to-sink remobilization of nitrate by mediating the degradation of *NRT1.7* in *Arabidopsis* (Liu et al., 2017). In the last decade, numerous studies suggested that the use of advanced soil and agronomical practices with modern molecular techniques could be the best way to enhance NUE swiftly under future climate change. Therefore, the objective of the present study is to discuss the role of advancement in agronomy, breeding, and molecular biology to enhance NUE and this information might be helpful in reducing N pollution.

## Functions of Nitrogen Throughout the Plant Life Cycle

In healthy plants, nitrogen concentrations range from 3 to 4%. When compared to other nutrients, this is a substantially higher amount. In plant cells, nitrogen is a significant source of various morphological, genetic, and metabolic components. The biological combination of nitrogen with C, H, O, and S to form amino acids, the building blocks of proteins, makes nitrogen a key component of protein (Okumoto and Pilot, 2011). Protoplasm, the site of cellular division and consequently of growth and development of plants, is formed using amino acids. The N increases the leaf area (AF) and leaf area index (LAI), promoting the synthesis of proteins involved in cell development, cell proliferation, and the development of the cell wall and cytoskeleton (Luo et al., 2020; Sun et al., 2020). Plant development and grain output are fueled by nitrogen’s role in the chlorophyll molecule, which allows the plant to absorb sunlight energy through photosynthesis (Evans and Clarke, 2019). Compounds that transport energy, such as ATP (adenosine triphosphate), include nitrogen. Adenosine triphosphate (ATP) helps cells store and utilize the energy supplied during metabolism (Evans and Clarke, 2019). As an osmotic agent N also plays an important role in water retention in plant vacuoles found to be essential toward its nutrition function. Water is the key limiting element in plant development since it is the main factor that can regulate cell growth and metabolism (Ding et al., 2018). Nitrogen raises the protein concentration of forage crops, improves the quality of the fruit, and speeds up the growth of green vegetables. Potassium and phosphorous are absorbed and utilized more efficiently by plants that are fed this nutrient (Chand et al., 2022). Nitrogen is essential for plants’ healthy growth and development. Nitrogen deficiency greatly impacts crop productivity, whereas extra N might have detrimental consequences on the plant. This topic is always being discussed in crop production. Therefore, this concern is constantly being addressed in agricultural output (Mu and Chen, 2021).

## Nitrogen Use Efficiency

As of today, the primary strategy for preserving and increasing agriculture production is the administration of mineral fertilizers, such as nitrogen. Commercial fertilizers contain N which is highly soluble, making it easy for plants to absorb and assimilate. Only 30–40% of the applied nitrogen is utilized by crops since the N compounds are often available in the form of nitrate and ammonium and are highly mobile in the soil (Omara et al., 2019). NUE can be described in a variety of approaches, but the most fundamental is the yield (grain, fruit, or forage) per unit of nitrogen present in the soil. The utilization of nitrogen by plants occurs in two distinct stages (Dobermann, 2005). In the first step, the quantity of N taken in, stored, and converted into amino acids and other essential nitrogenous molecules is a factor in the production of biomass. The eventual yield is determined by the amount of nitrogen (N) given to the seed at the second phase. Plants use the nitrate acquisition pathway to take in and synthesize inorganic nitrogen and store amino acids in immature leaves and roots. Their use in protein and enzyme biosynthesis, as well as the photosynthetic machinery that controls plant growth, architecture, and development, is extensive. For optimal flowering and grain development, nitrogenous molecules must be supplemented during the reproductive stage. Nitrogen absorption and remobilization are crucial, with leaves and shoots serving as a source of amino acids for the reproductive and storage organs (Chen et al., 2020). As a result, improving NUE necessitates an understanding of the processes by which N is taken up, assimilated, and remobilized throughout the plant life cycle. But over 60% of soil nitrogen is lost via leaching, runoff, denitrification, evaporation, and microorganism utilization (Ding et al., 2021). The intricacy of NUE lies in the multiple N sources that contribute to crop production, including soil N availability, conversion, accumulation, transport, and depletion, microclimatic conditions, crop genetics, and the effect of administration, environment, and climate (Chen et al., 2020). This leads to N losses in diverse types to the soil, air, and water, which is both economically and environmentally problematic.

## Nitrogen Uptake and Transport in Plants

Nitrate is a key source of nitrogen for plants; in fact, the majority of plants spend a substantial percentage of their carbon and energy reserves on its uptake and assimilation (Kant, 2018). Nitrate is a nutrient and a signaling molecule that has a dramatic influence on plant metabolism and development. Plants have evolved complex methods for detecting nitrate and integrating its absorption into their transport systems (Vega et al., 2019). Nitrate assimilation begins with its absorption into the cell. Normally, nitrate is taken up from the soil solution through the root’s apopalsm and then absorbed into the epidermal and cortical cells. Once inside the symplast, it is reduced or mobilized into the xylem for transport to the shoots through the Casparian strip (Islam, 2021).

**FIGURE 1:**
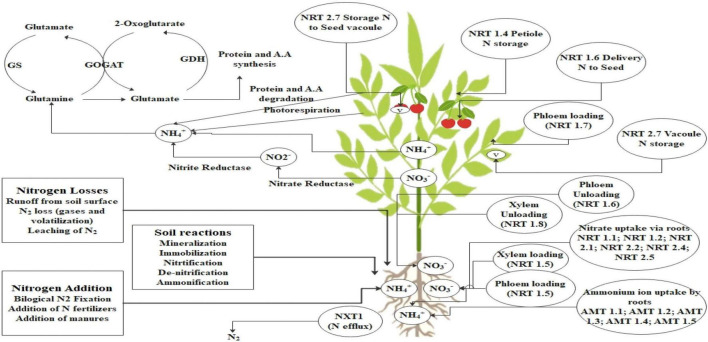
N dynamic in plants. This figure represents the addition and losses of N through soil. There are several soil reactions such as mineralization, nitrification involved in the conversion of fixed N to available form and then uptake and transport from root to shoot. Nitrate (NRT; nitrate transporter) and ammonium (AMT; ammonium transporter) are the major forms of N movement through soil to root, root to root-shoot junction, then through phloem tissue. In this process, several transporters are involved which are highlighted in the figure such as phloem loading and unloading, xylem loading and unloading. After reaching the N in the leaf tissue is used for conversion of different compound synthesis and stored in the form of amino acids and proteins.

Plants utilize both high affinity and low-affinity transporters to import nitrate. These import pathways allow plants to accommodate a wide range of external nitrate concentration without experiencing severe deficiency or toxicity. A wide array of genes are associated with the uptake of nitrogen in plants. The nitrate uptake by plant roots appears to be a complex process involving at least four distinct transport systems: (a) constitutive high-affinity transporters (cHATS); (b) nitrate-inducible high-affinity transporters (iHATS); (c) constitutive low-affinity transporters (cLATS); and (d) nitrate-inducible low-affinity transporters (iLATS) (O’Brien et al., 2016). In plants, two families of genes producing nitrate transporters have been identified: *NRT1* and *NRT2.* The NRT 2 family encodes transporters to the high-affinity uptake system whereas NRT 1 family is more complex, including nitrate transporters with dual affinity or low affinity. The nitrate uptake is driven by the proton gradient against the plasma membrane and its uptake is driven by ATPases (Wang et al., 2020; You et al., 2022). In comparison to nitrate, plants generally do not collect excessive amounts of ammonium ions. Toxicity symptoms usually occur when agricultural plants are cultivated in ammonium deficient in nitrate (Duan et al., 2018). Initially, a family of five ammonium transporter genes named AMT1;1–AMT1;5 was found in *Saccharomyces cerevisiae* and *Escherichia coli* (Loqué and von Wirén, 2004). However, the scenario is somewhat different in rice, where 10 distinct genes have been found (Suenaga et al., 2003). Attempts have been made further to identify transporters of nitrogen uptake in plants and overexpression of nitrogen transporters genes will lead to enhanced nitrogen uptake efficiency of the roots from the soil. Some of the nitrogen and ammonium transporters and their function are highlighted in Figure 1.

## Advances in Agronomical Strategies to Enhance Nitrogen Use Efficiency

### Advancement in Fertilization

**FIGURE 2:**
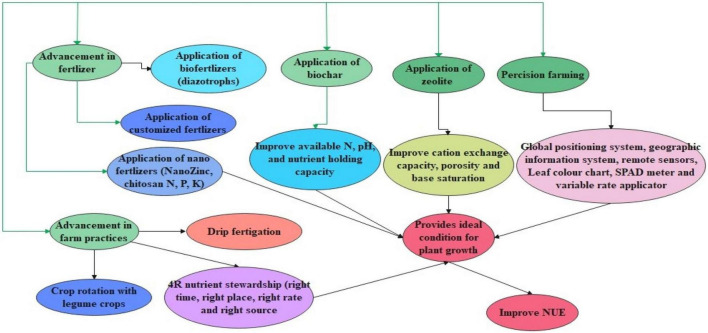
Represents the advancement in agronomical practices for improving NUE. This figure highlights the advances such as the addition of nano fertilizers, customized, and bio-fertilizers which improve the NUE. Further, the addition of biochar and zeolites is also helpful in improving NUE through improvement in soil properties. Together with the advancement in farm practices such as drip fertigation, crop rotation, and best management improve NUE. Also, the application of advanced technology such as remote sensing has a bright future in improving NUE.

Fertilizers play a pivotal role in enhancing and sustaining crop yield. It is reported that a 50% yield increment is contributed by nitrogen (N) fertilizer. However, about 40–50% of the applied N is lost by ammonia volatilization, leaching, run-off, and denitrification. Therefore, in place of conventional chemical fertilizers, the use of modern fertilizers could be a potential alternative (Dass et al., 2017). These fertilizers are more efficient than conventional fertilizers to meet the food requirement of the burgeoning population and improve NUE, it is important to look for new fertilizer materials that help ensure world food security on one hand and safeguard the environment on the other (Singh et al., 2019). Some of the advanced strategies in improving NUE are highlighted in Figure 2.

#### Customized Fertilizers

Customized fertilizers (CF) are a multi-nutrient carrier that contains macro and micro nutrient forms (Mudalagiriyappa et al., 2015). “Paras Formula” was the first CF developed in India which contains (10% N, 18% P_2_O_5_, 25% K_2_O, 3% S, and Zn 0.5%). It encompasses both macro and micro-nutrients required by selected crops in specific regions. Adoption of such fertilizers would enhance NUE which is currently 40% for N, 20% for P, 50% for K, and 2–5% for other micronutrients (Dass et al., 2017). Many scientists have reported that the application of CF improved the yield, NUE, nutrient uptake, and was also more economical. Sekhon et al. (2012) observed that application of CF (16% N, 24% P, 9% K, 5% S, and 0.7% Zn) in wheat crop increased the growth, grain yield, agronomic efficiency of N, and economics in Ludhiana region of Punjab. Similarly, Dwivedi and Meshram (2014) also reported that application CF (11% N, 18% P, 9% K, 5.3% S, and 0.7% Zn) in wheat improved uptake of N, P, K, and Zn which ultimately crop led to higher grain yield under Raipur region of Chhattisgarh. In Maharashtra also use of CF (20% N, 12% P, 10% K, 4% S, 0.25% Mg, 0.5% Zn, and 0.5% Fe) in onion improved the soil fertility as well as economics (Kamble and Kathmale, 2015). The quality of pomegranate has been enhanced through the application of CF (20% N, 10% P, 10% K, 5% S, 2% Mg, 0.5% Zn, 0.3% B, and 0.2% Fe) in the Rahuri region of Maharashtra (Goel et al., 2011).

#### Nano Fertilizers

Due to the subsidization of fertilizers, farmers apply fertilizers in an excess amount which ultimately either enters into the groundwater causing nitrate pollution, or into aquatic systems leading to eutrophication (Shah et al., 2021a). To sort out the problem of excess consumption of fertilizers, enhance the NUE, and prevent environmental hazards nano-fertilizers (NFs) could be a great alternative and used by regulating the release of nutrients depending on the requirement of crops (Mejias et al., 2021). Application of NFs enhanced the productivity of crops through the slow and continuous release of nutrients to the roots of plants and thereby increasing NUE (Kah et al., 2019). Application of Zn NFs improved the rice grain yield by increasing NUE through alteration in physiological characteristics (Jin et al., 2017). Application of engineered nanomaterials made up of amorphous pyrogenic hydrophilic SiO_2_ influenced the availability of P by increasing its mobility. It also boosted the NUE by decreasing the demand for P and N fertilizer for the growth of plants. Foliar application of NFs (chitosan-NPK) enhanced the productivity and nutritional status of wheat (Abdel-Aziz et al., 2016). Nano Zn-chitosan in wheat crop applied as soil application (20 mg g^–1^ soil) enhanced the accumulation of Zn in plants grown under Zn deficient soil (Deshpande et al., 2017). Soil application (6 mg kg^–1^ soil) of nano-ZnO in sorghum enhanced the productivity, uptake, and NUE (Dimkpa et al., 2017). Application of nCeO_2_ in barley crop improved the Ce concentration in grain, as well as uptake of macro- and micro-nutrients (Rico et al., 2015). Similarly, soil application of nano-titanium oxide (nTiO_2_) to tomato plants improved the growth, uptake, and accumulation of plant nutrients (Raliya et al., 2015).

#### Bio-Fertilizers

Bio-fertilizers are formulations of beneficial microorganisms which directly or indirectly enhance microbial activity and thereby increase movement and solubilization of nutrients in the soil (Suyal et al., 2016). In the case of direct mechanism, diazotrophs (*Azotobacter*, *Azospirillum*, *Acetobacter*) and phosphate solubilizing bacteria (PSB) improve the plant growth by producing phytohormones, liberating nutrients, and stimulating induced systemic resistance (Rani et al., 2013). Under indirect mechanisms, *Bacillus*, *Pseudomonas*, and *Mycorrhiza* enhance plant growth by legume symbioses by stimulating symbiotic relationships for root growth (Podile and Kishore, 2006). Diazotrophs have the capacity to reduce N_2_ to ammonia, and flora and fauna rely on biologically fixed nitrogen for growth and development (Kumar et al., 2014). PSB have the capacity to solubilize the unavailable P into available form up to 50% (Chen et al., 2006). Mycorrhiza has the potential to go deeper in the soil and provide the available form of nutrients to the plant roots thereby increasing the uptake (Mallik, 2000). Bio-fertilizers help in the mineralization of nutrients through the decomposition of organic matter and ultimately improve soil fertility. They also play important role in immobilization by converting atmospheric N to NH_4_^+^ for plant uptake (Paul, 2014). Bio-fertilizers are an important component of soil and crop management. They act as organic amendments by decomposing the crop residue, managing the deleterious soil insect-paste, and ultimately improving the soil resilience and sustainability of the agriculture production system (Sahoo et al., 2013).

#### Biochar

Biochar is a charcoal-like material produced from partial pyrolysis of organic material produced from agriculture, increasing the nutrient availability and organic carbon in the soil ultimately soil fertility and NUE (Majumder et al., 2019). Biochar is attributed with a high surface area, pores, and different functional groups which imparts nutrient holding capacity in soil (Prommer et al., 2014). Biochar has great potential in improving the soil pH. Similarly, it also improves the aeration and moisture retention capacity, porosity, and microbial density of the soil. The combined use of wheat residue and biochar along with nutrients at 1 and 2% doses improved the available N and pH of the soil (Mierzwa Hersztek et al., 2020). The use of biochar produced from woody components significantly improved the cation exchange capacity of soil owing to the oxidation of specific functional groups on the surface of biochar (Cornelissen et al., 2013). Recalcitrant propriety of biochar prepared from rice husk improved the soil organic carbon content in soil (Jatav and Singh, 2019). The application of biochar not only enhanced the productivity of crops but also the diversity and density of beneficial micro-organisms in soil (Azeem et al., 2020a). It has also been observed that the combined application of compost and biochar enhanced the urease, dehydrogenase, and β-glucosidase activity in the soil (Azeem et al., 2020b).

#### Zeolites

Zeolites are a group of naturally occurring minerals (aluminosilicate) that consist of distinct chemical compositions. Clinoptilolite is the most widely found zeolite. It is attributed with high cation exchange capacity, surface area, base saturation, and porosity which make it a potential candidate for improving the chemical properties of soil (Gholamhoseini et al., 2012). Zeolites have a minute void diameter (0.3–0.8 nm) that helps in the fixation of NH^4+^ ion and slow release of N which ultimately minimizes the N losses that occur through volatilization and denitrification. The main use of zeolites is for N capture, storage, and slow-release, as they adsorb molecules at relatively low pressure and are considered as a nano-enhanced green application (Lavicoli et al., 2014). It has been found that the application of zeolites in the field improved crop productivity and NUE (Ramesh and Reddy, 2011). Kavoosi (2007) found that application of 16 tons zeolite ha^–1^ increased apparent N recovery up to 65%, agronomic efficiency up to 22 Kg grain kg^–1^ N, and ensured good retention of soil-exchangeable cations, available P, and NO^3–^ within the soil in maize at Malaysia (Rabai et al., 2013). The application of zeolites along with rock phosphate tremendously increased the uptake of P by crop plants through exchanged induced dissolution (Yuan et al., 2008). Surface application of zeolite has the potential for mitigating NH_3_ losses thereby reducing losses of nitrogen to the environment (Waldrip et al., 2014). Ammonium-charged zeolites have shown their ability to increase the solubilization of phosphate minerals, promote rock phosphate dissolution in all soil types, and reduce fixation in soils (Shokouhi et al., 2015).

### Role of Precision Farming in Improving Nitrogen Use Efficiency

Precision farming is an information and technology oriented farm input handling approach that aims to identify, analyze, and manage spatial and temporal variation present within the field by performing all practices of crop production in right place at right time in the right way for optimum profitability, sustainability, and protection of land resources (McBratney et al., 2003). Global positioning systems (GPS), geographic information systems (GIS), remote sensing, and variable rate applicators (VRA) are the technologies that play important roles in the efficient application of nutrients. Further, different precision farming tools like, leaf color chart (LCC), chlorophyll meter, and green seeker are also playing an instrumental role in increasing NUE (Dass et al., 2015a).

#### Remote Sensing Technologies

The GPS provides location information in real-time while it is moving. After getting specific location details, the collection of soil and crop location data could be accomplished efficiently. GPS receivers can be kept along with farm implements, which permits the users to return to precise locations for managing those areas. GIS are computer systems that utilize features, characters, and location data for obtaining maps. They also store remotely sensed data and soil nutrient levels. Remote sensors of N are managed in an experienced way for evaluating the demand for N to be fertilized into the cropping system (Schmidt et al., 2002). In sensor oriented approach a non-exhaustive N band is kept within the field which acts as a control or reference. Sensors have the capacity to assess whether a crop requires extra N by postponing the application of some amount of N normally used (Pathak and Ladha, 2011). Remote sensing techniques collect data from distance. Remote sensing data provide a tool for determining moisture, nutrient, and health stress which are captured in overhead images. It has been reported that plant N level and normalized difference vegetation index (NDVI) have a strong correlation. The NDVI increases with increasing leaf greenness, therefore, remote sensing could be used a toll for N application (Gandhi et al., 2015). VRAs have three components namely the computer, locator, and actuator. The computer utilizes the application map and GPS send a signal to the input controller which modifies the amount of input to be applied in the field. The NUE in wheat increased by 15% when fertilization was accomplished based on optical VRA techniques (Raun et al., 2002).

#### Leaf Color Chart

A leaf color chart (LCC) is used for evaluating the leaf N concentration based on the chlorophyll content of the leaves (Singh et al., 2010). It is a diagnostic tool that can be used to optimize the N requirement and nutrient management in the rice-wheat cropping system (Yadav et al., 2017). The LCC reading is taken 10 days after sowing or 20 days after transplanting to heading. An LCC value of 3 and 4 is critical for basmati and hybrid rice, respectively.

Application of N through an LCC-based approach in hybrid rice resulted in a 25% saving of N fertilizer without compromising crop yield (Singh et al., 2012). It has been reported that the highest NUE and recovery efficiency of applied N was found with a 120 kg recommended dose of nitrogen in rice at an LCC value of 4. LCC-based nitrogen application was found to be effective in improving rice yields with limited nitrogen supply on a plant-need basis (Bhavana et al., 2020). Similarly, partial factor productivity (52.9 kg kg^–1^), NUE (19.2 kg kg^–1^), and RE (54.3%) were found higher under N followed the management with LCC in different rice genotypes in Gujarat. Application of N at LCC4 was found better than LCC5 and LCC3 in terms of yield and nitrogen saving (Gudadhe and Thanki, 2021).

#### Chlorophyll Meter

Soil Plant Analysis Development (SPAD) is also called a chlorophyll meter that measures the chlorophyll concentration of leaves. It is used to determine the effectiveness of top-dressed N for improving the productivity and protein concentration in the crop plants (Singh et al., 2012). The application of N in a maize crop according to a chlorophyll meter (SPAD value ≤ 37) approach led to a saving of 55 kg N per hectare without affecting the yield of the crop. It has been reported that NUE was found highest with SPAD-based (≤37) N application and the lowest with soil-based N application (Dass et al., 2015b). Similarly, in the rainy season maize results showed that, applying N based on SPAD value < 37.5 recorded the highest grain yield (5.2 t/ha) which was significantly higher than soil-based and SPAD value < 35 based N application. Water productivity (10.4 kg/ha-mm) and agronomic efficiency (26.0 kg/kg N) were also the highest with this treatment (Dass et al., 2014). Precise application of N in wheat crops based on chlorophyll meter at SPAD value of ≤ 42 led to increased crop productivity by as well as saving of 20 kg N per hectare (Dass et al., 2012).

### Advances in Agronomical Practices

#### Diversified Crop Rotation

Diversified crop rotation is one of the major components of conservation farming. It has a tremendous role in optimum utilization of resources and enhancement of resource use efficiency. Adoption of diversified crop sequences ensures precise application of inputs (nutrients and moisture) to crops for maintaining the sustainability of the agricultural production system (Singh et al., 2012). Inclusion of summer mungbean (SMB) in the maize-wheat cropping system after harvesting of wheat improves the NUE and soil fertility by fixing free atmospheric N and reduces the N requirement of the succeeding crop gown in the sequence (Parihar et al., 2017). Growing aerobic rice instead of transplanted rice leads to saving irrigation water as well as the N requirement of the crop by curtailing the denitrification, ammonia volatilization, and leaching of N as occurs in transplanted rice (Jinger et al., 2020). Further, it also enhances the NUE of applied nutrients under aerobic rice (Jinger et al., 2021). Zero-till wheat followed legume with residue retention has great potential to add organic matter and N into the soil which eventually increases the water as well as NUE (Das, 2014).

#### 4R Nutrient Stewardship

The term “4R” refers to the use of the right source, right rate, right time, and right place in nutrient management (Fixen, 2020). 4R nutrient stewardship is the key to achieving balance fertilization and higher NUE in the cropping system. To realize the maximum benefit of every nutrient management practice the identification of 4R nutrient stewardship for each nutrient and in every crop is crucial (Bruulsema et al., 2019). The right source matches the fertilizer types to crop needs. Yang et al. (2016) has reported that modification in N source leads to a reduction in N losses. Application of specially enhanced efficiency compounds like nitrification or urease inhibitors delayed the N transformations and minimized its losses. Another method is correctly matching the amount and delivery rate of fertilizer to crop needs. Sela and Harold (2018) reported that optimal N rates rely on soil texture, mineralization rates, losses, and sources of N inputs. The right time means making nutrients available when the crop needs them. Under irrigated conditions, N fertilizers should be applied in two to three equal splits. In medium and heavy soils two-thirds or half of the dose should be applied at the time of sowing and the remaining one-third or half-dose should be top-dressed at first irrigation (Dhar, 2014). The right place means keeping nutrients where crops can use them efficiently (Flis, 2018). Banding of N in the soil through injectors can also increase N availability by applying the product closer to the crop roots (Westerschulte et al., 2017).

#### Drip Fertigation

The application of nutrients to crops through a drip system is called drip fertigation. It is the most advanced method in nutrient as well as in water management. Farmers are practicing drip fertigation, particularly for winter wheat, across the globe owing to significant improvement in WUE and NUE. Drip fertigation matches the water and N supply with crop demand which eventually enhances water productivity and NUE (Si et al., 2020). It has been revealed that drip fertigation improved the crop yield, WUE, and NUE. Further, drip fertigation led to a reduction in evapotranspiration significantly as compared to flood irrigation and broadcasting method of N application (Li et al., 2021). Sub surface drip fertigation system has an instrumental role in saving irrigation water, energy and in increasing NUE (Sidhu et al., 2019). PFPN decreased with the increase in the N application rate through drip fertigation. The highest nitrogen production efficiency (264.4 kg/kg) and partial factor productivity (265.9 kg/kg) of N in cucumber were recorded under drip fertigation levels of 80 and 100% reference evapotranspiration, respectively (Wang et al., 2019).

#### Crop Modeling

Crop models are a collection of mathematical equations that were developed for predicting plant growth and development in precise ways (Kephe et al., 2021). Also, they have shown promising results in the decision support system, and policy development under climate change scenarios. There are a number of models developed for the assessment of NUE in crops. For instance, in northeast China, DeNitrification-DeComposition (DNDC) and Decision Support System for Agro-technology Transfer (DSSAT) were developed for exploring management strategies to stimulate yield and NUE in maize crops. Further, from 7 years of experimenting they suggested that the application of these models helps in adjusting the fertilizer rates and time and planting density and dates, which are associated with improvement of NUE (Jiang R. et al., 2019). Likewise, the NDICEA (Nitrogen Dynamics In Crop Rotations in Ecological Agriculture) crop model describes the soil water, organic matter, and N dynamics in relation to crop demand and weather conditions. The implication of this model was that the application of combined inorganic and organic fertilizers improve the crop yield, and NUE (Van der Burgt et al., 2006). The SPACSYS model was used for stimulating crop yield and NUE in wheat and maize under climate change. This study results suggested that climate change reduces the NUE by 15% in north China, which can be compensated by the advanced soil management practices and higher application of N, P, K, and manure (Liang et al., 2018). Consequently, numerous studies conducted in diverse agricultural crops state that the adoption of effective management strategies combined with the crop models enhance the NUE and yield attributes.

## Cross Talk of Other Mineral Nutrients With Nitrogen and Effect on Nitrogen Use Efficiency

Nitrogen is the major essential nutrient required by the plants and it interacts with almost all the other essential nutrients. N has synergistic interaction with most of the nutrients that leads to enhanced NUE. However, the kind of interaction depends on the number and amount of interacting nutrients present in the soil and their application rate. If both the interacting nutrients have an imbalanced amount in the soil it could cause antagonistic interaction (Datta and Meena, 2015). Jiang J. et al. (2019) reported that P x N have positive interaction (synergistic) because the application of P improved the N content in the plant. Dwivedi et al. (2003) also observed that combined fertilization of N and P improved the NUE by 28% in wheat crop. Conversely, Aulakh and Malhi (2005) observed that in P deficient soil, sole application of N led to a reduction in grain yield and reported antagonistic interaction. Barker and Pilbeam (2015) found that fertilization of K enhanced the N uptake and assimilation in plants, and eventually NUE, by many folds. However, they also reported that when K is low in the soil, it competes with NH4 for selective binding sites in the adsorption process and eventually caused an antagonistic effect. Sulfur is the most important secondary nutrient, and Jamal et al. (2010) revealed that S application has an instrumental role in enhancing the recovery efficiency and NUE of applied N. However, Rietra et al. (2017) reported that in S deficiency condition, application of N leads to abundant accumulation of a pernicious level of N metabolites in plants. Wilkinson et al. (2000) concluded that Ca and Mg enhanced solubility of fixed N in the acidic soils, and improved N translocation in plant. However, in Mg deficient soil, sole application of N fertilizers causes grass tetany in livestock caused by low Mg concentration in forage. N fertilization improved the Zn absorption, translocation, and assimilation in crops and led to increased zinc use efficiency and vice versa. Antagonistic effect of N fertilization on Zn nutrition either due to dilution effect (decrease or dilution in plant nutrient concentration due to increase in biomass yield) effect or poor translocation of Zn-protein complex in the roots. The application of N fertilizers enhanced the availability of Fe and Mn by increasing acidity which further led to the conversion of unavailable forms of Mn and Fe into available forms (Aulakh and Malhi, 2005). In optimum conditions, N has a positive interaction with Cu but in the case of Cu deficient soil application of N resulted in crystal clear symptoms of Cu deficiency in plants (Barker and Pilbeam, 2015). The interaction effect of B, Cl, Mo, and Ni with N has not been reported so far. However, some studies reported that these nutrients are helpful in increasing NUE. In acidic conditions Al and Ce reduced the uptake and assimilation of N due to reduced growth of roots ultimately decreased NUE (Hille et al., 2011; Zhao and Shen, 2020). The cross-talks of different mineral elements with N are highlighted in Table 1.

**TABLE 1:** Cross talk of N with other essential elements.

Mineral nutrient	Effect of N concentration/uptake	Mechanism	References
P (Optimum)	Enhance N concentration in plant	Synergistic	Jiang J. et al., 2019
P (Deficient)	Application of N alone could cause a severe reduction in grain yield	Antagonistic	Aulakh and Malhi, 2005
K (Optimum)	Increase NH_4_^+^ assimilation in plant	Synergistic	Barker and Pilbeam, 2015
K (Deficient)	Competes with NH_4_ for selective binding sites in the adsorption process	Antagonistic	
S (Optimum)	Increased recovery and NUE	Synergistic	Jamal et al., 2010
S (Deficient)	Excessive accumulation of toxic levels of N metabolites in the plant	Antagonistic	Rietra et al., 2017
Ca (Optimum)	Increased water-soluble N and fixed NH_4_ in the acidic soils, leading to increased N uptake	Synergistic	Wilkinson et al., 2000
Mg (Deficient)	Application of N fertilizers cause grass tetany in livestock caused by low mg concentration in forage	Antagonistic	
Zn (Optimum)	Nitrogen improved Zn absorption by plants and vice versa.	Synergistic	Datta and Meena, 2015
Zn (Deficient)	Antagonistic effect either due to dilution effect (decrease or dilution in plant nutrient concentration due to increase in biomass yield) effect or poor translocation of Zn-protein complex in the roots.	Antagonistic	
Fe	Application of N increased acidity with NH_4_ may enhance the availability of Fe^2+^ by promoting the reduction of Fe^3+^.	Synergistic	Aulakh and Malhi, 2005
Mn	Application of N leads to reduction of the unavailable Mn^4+^ to available Mn^2+^ in soil	Synergistic	
Cu (Deficient)	Cu deficiency symptoms became more severe when N was applied to Cu deficient soils.	Antagonistic	Barker and Pilbeam, 2015
Al	Al inhibit root growth and uptake of N	Antagonistic	Zhao and Shen, 2020
Ce	Decrease N assimilation	Antagonistic	Hille et al., 2011

## Advances in Physiological Approaches for Improving Nitrogen Use Efficiency

**FIGURE 3:**
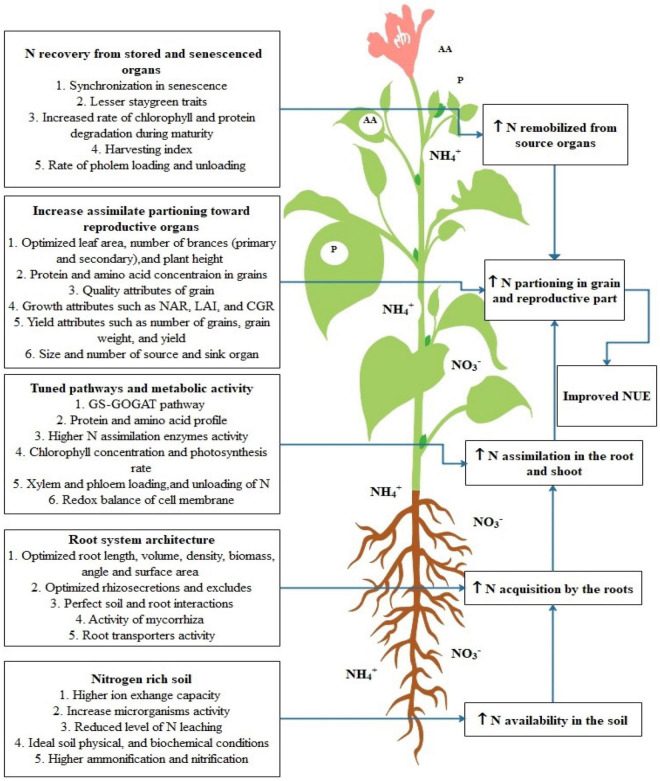
Highlights the crucial plant traits for improvement of NUE in plants. In this figure first process includes the traits which are crucial for increasing N availability in soil, then most of soil properties and reactions are associated with this. Further, this increased need for N acquisition by the root tissue for this root architecture traits plays an important role. Later on, the biochemical processes in leaf tissue contributes to N assimilation in root and shoot tissues. Consequently, the N remobilization from stored tissues is crucial for increasing NUE and the last N portioning is the next crucial process that determines the N availability in reproductive tissues.

Physiological attributes play a pivotal role in improving NUE in plant species. Among those attributes, the root system is crucial in the acquisition of water and nutrients through its ability of soil exploration which is the main determinant of NUE (Li X. et al., 2016). NUE depends upon the root distribution in soil, root size, and root/shoot (R/S) ratio (Garnett et al., 2009). These factors maximize the uptake and interception of nitrogen as well as reduce the nitrogen losses to groundwater and deeper layers of soil (Lynch, 2013). The important plant attributes related to N uptake, acquisition, assimilation, remobilization, and portioning are highlighted in Figure 3. Various research studies have been conducted to show that root system architect (RSA) is closely related to the NUE e.g., plants having deeper and steeper roots can uptake nitrogen effectively from deeper soils (Zhan and Lynch, 2015). A research study on two varieties of Chinese (XY335 and ZD958) and one variety of US (P32D79) maize was conducted to analyze the effect of the root system on NUE. Root analysis with respect to NUE revealed the positive correlation of NUE with dry weight (RDW) and root/shoot (R/S) biomass ratio. While RDW and R/S ratio of western variety (P32D79) were greater than Chinese varieties (XY335 and ZD958) similarly, it was also observed that P32D79 had a better root system which leads to higher N-uptake and removal of more N-minerals than Chinese varieties from the soil. Thus, it was concluded that maize variety with a better root system and higher stress resistance underlines higher NUE (Yu et al., 2015).

Due to the major role of the root system in NUE, its genetic basis is also subjected to study. A research study was conducted to understand the genetic association between root traits of maize seedlings and NUE through QTL analysis. Recombinant inbred lines of maize were sown under low and high nitrogen conditions in which 9 traits of RSA and 10 traits of NUE were evaluated in three hydroponic and four field conditions, respectively. A significant correlation was observed between uptake efficiency of nitrogen and RSA (especially in the crown and seminal roots). Almost 331 QTLs in total were identified including 147 QTLs of RSA and 184 QTLs of NUE. Almost 70% of NUE-QTLs coincided with the QTL clusters of RSA which indicated the significant genetic association between traits of NUE and RSA. This association can aid in the selection of RSA-traits for improvement of NUE in the maize (Li et al., 2015).

In a research study, recombinant inbred rapeseed “BnaZNRIL” was studied to investigate traits related to NUE and root morphology (RM) under high and low nitrogen hydroponic conditions. Results indicated the significant correlation of root size (trait related to RM) with N-uptake and dry biomass of plant while no correlation was observed with the efficiency of nitrogen utilization (NUtE). Approximately 23 stable QTLs out of a total of 129 were detected in both high and low nitrogen conditions. Most of the stable QTLs (20/23) were observed to be related to RM traits under both high and low nitrogen conditions which lead to the suggestion of regulating the root morphology through NUE is more feasible than through regulating the nitrogen efficiency in rapeseed (Wang et al., 2017). Another genome-wide association study was conducted to understand the effect of low nitrogen levels on root growth and root traits at seedling emergence level. A panel of 461 inbred lines of maize was assayed for the root growth under low nitrogen (0.05 mml^–1^) and high nitrogen level (5 mml^–1^). The panel was further genotyped with an SNP marker of 542,796 high density. Various root traits were observed to be increased under low nitrogen conditions such as total root length, dry weight of root, root-to-shoot ratio, lateral and axial root length of the primary root. Furthermore, a wider heritability range of 0.43–0.82 was observed under low nitrogen conditions than the range of 0.25–0.55 under high nitrogen conditions. After marker-assisted analysis, it was observed that under low nitrogen conditions, gene encoding DELLA protein was in association with the lateral root-zone of primary root and *protoporphyrinogen IX oxidase-2* gene was in association with the surface area of the root. While under high nitrogen conditions the histone-deacetylase gene was in association with the plant height which can be further used to exploit genetic improvement in root traits through better NUE of maize (Sun et al., 2021). The important root traits and associated genes in response to NUE are highlighted in Table 2.

**TABLE 2:** Major quantitative trait loci (QTL) associated with NUE in cereals crops.

Crop	QTLs	Chromosome no.	References
Rice	qNUE2.1	2	Zhou et al., 2017
	*qNUP3.1*	3	
	qNUE4.1	4	
	qNUE6.1	6	
	qNUE6.2	6	
	*qNUP8.1*	8	
	qNUE10.1	10	
	qNUE10.2	10	
	*Qnr*	1	Nguyen et al., 2016
	*QaNUE*	8	
	*qNAA4*	4	Dai et al., 2015
	*qNAA5*	5	
	qNAA10	10	
Wheat	*QNue.151-1D*	1D	Brasier et al., 2020
	*QNue.151-4A*	4A	
	*QNue.151-6A*	6A	
	*QNue.151-7D*	7D	
	*NutE2*	3A	Cormier et al., 2016
	NUE8	1A	
	NUE10	3A	
	NUE2	3B	
	NUE2	3B	
Barley	*qNUEg*	2H	Kindu et al., 2014
	*qNUEb*	3H	

## Advances in Conventional Breeding for Improving Nitrogen Use Efficiency

Cereals, such as rice, wheat, and maize, are the most important sources of calories and nutrition for humans. Modern varieties differ in key NUE characteristics (Cormier et al., 2013; Barraclough et al., 2014). However, it is clear that a broader germplasm base has a much higher potential for variation (Monostori et al., 2017). The efficient use of resources, including fertilizers such as nitrogen, is critical to long-term sustainability. According to Raun and Johnson (1999), globally only 33% of applied nitrogen fertilizer is recovered in harvested grain. The main problem with landraces and relatives is that while biomass may be high, yields and in particular HI are normally low, making traditional NUE measurements less useful. Height, which is influenced by dwarfing genes, is the most important architectural influence. While Rht genes may have a negative effect on height, they may also have other pleiotropic effects, such as decreased root proliferation (Gooding et al., 2012; Bai et al., 2013). The efficiency of absorption will be aided by variations in root architecture and function. Root traits have been deconstructed as quantitative trait loci (QTL) in a number of studies. The variation in root proliferation, length, lateral profusion, and spread or angle of roots has been identified (Gooding et al., 2012; Atkinson et al., 2015). Wheat has a large number of nitrate transporter genes that are involved in both initial uptake and internal translocation mechanisms.

NUE is a polygenic trait with a number of factors. As a result, identifying individual genetic effects involves quantitative genetics strategies, and such effects have been usually characterized as QTL. There are several methods for identifying genes that control NUE and utilizing them in breeding. Associations genetics could be based on genetic panels that are well-suited to the researcher’s test environment, sample multiple alleles, and provide extremely high genetic resolution. Furthermore, association genetics requires a good balance of alleles at each location analyzed, with low-frequency alleles (usually less than 10%) being excluded from the research. Segregating populations derived from two diverse parents is the best-established way of QTL identification. Recombinant Inbred Lines and Single Seed Descent or the doubled haploids are statistically strong because only the two parental alleles are segregating at anyone locus and the population is comprised 50% of each allelic class.

A number of studies on NUE QTLs have been well known by this approach for example in rice (Wei et al., 2011). Multi Parent Advanced Generation Intercross (MAGIC) permits the concurrent analysis of several alleles and the mapping resolution afforded by recombination of the population as well as historical recombination (Mackay et al., 2014). Wingen et al. (2017) produced a publicly available NAM population that represents more than 90 landrace parents and over 10,000 recombinant inbred lines. Preceding studies of cereal crops have searched for novel NUE traits and alleles in adapted breeding materials (Fontaine et al., 2009), landraces (Pozzo et al., 2018; Van Deynze et al., 2018), and wheat wild relatives (Hu et al., 2015). While these authors have successfully identified QTLs, genes, and genotypes conferring high NUE, additional sources of genetic variation likely still exist within the currently unexplored germplasm.

## Advance in Molecular Approaches for Improving Nitrogen Use Efficiency

**FIGURE 4:**
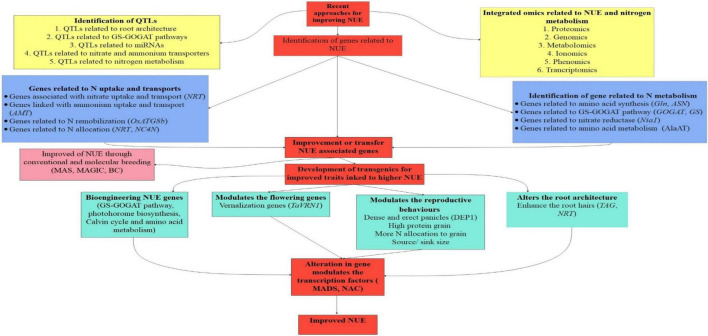
Represents the advances in molecular biology in improving NUE. In this, the identification of QTLs (Quantitative trait loci) related to root architecture, miRNAs (micro RNA), transporters, and metabolism is crucial for improving NUE. In this sequence the application of genomics, proteomics, phenomics, and metabolomics have great opportunities to enhance NUE. Then the identification and transfer of genes related to NUE and nitrogen metabolism play a crucial role in the development of high-NUE efficient plants.

Recently the development of cultivars with improved NUE is of utmost essential as after decades of green revolution application of N fertilizer is increased tremendously to meet grain yield demands. Simultaneously, its negative impact may be witnessed in the form of the increased cost of cultivation and hazardous effect on the ecosystem. The transcription factors, allosteric control, and post-transcriptional modification all play a vital role in the expression of a complex trait like NUE (Basu and Jenkins, 2020). Because quantitative traits like NUE are influenced by a large number of genes with minor effects and environmental influence, identifying QTLs for such traits necessitates a larger mapping population with phenotyping at a variety of locations and environmental conditions, as well as a sufficiently large coverage of the genome by the markers. Though, major QTLs with major and minor effects that can easily be incorporated in the breeding cycle are very limited in number. However, minor QTLs with minor effects are more in number but they require proper validation across the population. The molecular information on genes involved in various stages of the N metabolic process, from protein to final metabolites can aid in the identification of QTLs associated with NUE or its components. The important molecular biology tools for improving NUE are highlighted in Figure 4.

### N-Transporters/Genes/Quantitative Trait Locis Identified to Enhance Nitrogen Use Efficiency

Selection of genotypes having root structure association (RSA) based NUE is recommended in wheat due to a limitation of full comprehension regarding the genetic background of NUE and associated G × E interactions. Wheat QTL analyses for all RSA characteristics are also available. Numerous wheat QTLs for NUE attributes have been discovered at the physiological and agronomic levels (Fan et al., 2019; Brasier et al., 2020). Despite these considerable advancements in knowledge of NUE and RSA genetics, only a small percentage of these QTLs and related markers could be used in practical breeding (Saini et al., 2021).

A meta-QTL (MQTL) study appears to be a viable technique for overcoming the aforesaid constraints in employing QTLs for the production of wheat cultivars with greater NUE, as it permits identification of the most robust and stable MQTLs. Because MQTLs have lower confidence intervals (CIs) and better phenotypic variation explained (PVE %) than QTLs, they are more useful in marker-assisted selection (MAS). MQTLs have also been demonstrated to be effective in identifying interesting candidate genes (CGs) linked to the trait of interest. Already, this approach has been utilized in the identification of MQTLs in various major crops like wheat, rice (Khahani et al., 2020, 2021; Kumar and Nadarajah, 2020), barley (Zhang et al., 2017), and maize (Zhao et al., 2018). The physical position of these MQTLs was for NUE was confirmed by using the GWAS approach on durum (4x) and bread wheat (6x), results depicted that 45 MQTLs were confirmed for durum wheat and 81 for bread wheat while 38 MQTLs verified in both ploidy wheat (Saini et al., 2021). The agronomic nitrogen−use efficiency of rice can be increased by driving *OsNRT2.1* expression with the *OsNAR2.1* promoter (Chen et al., 2016). Moreover, altered expression of the PTR/NRT1 homolog *OsPTR9* affects nitrogen utilization efficiency, growth, and grain yield in rice (Fang et al., 2013). Previous research by Zhao et al. (2021) reported that *PtoNRT* genes exhibited distinct expression patterns between tissues, circadian rhythm points, and stress responses. The association study showed that genotype combinations of allelic variations of three *PtoNRT* genes had a strong effect on leaf nitrogen content. The weighted gene co-expression network analysis (WGCNA) produced two co-expression modules containing *PtoNRT* genes. Moreover, *PtoNRT* genes defined thousands of eQTL signals. WGCNA and eQTL provided a comprehensive analysis of poplar nitrogen-related regulatory factors, including *MYB17* and *WRKY21*.

Similarly, in maize plants, it has been reported by many authors that some gene domains/families which are responsible for other gene regulations were also found to be engaged with NUE. For example, kinase domain (98 genes), for protein-containing F-box like domain (79 genes), for cytochrome P450 proteins (40 genes), for glycoside hydrolases (32 genes), for UDP-glucosyltransferases (13 genes), for NAC TFs (16 genes), for expansions (14 genes), for early nodulin-93 proteins (13 genes), for GRAS TFs (10 genes), and ABC transporter proteins (10 genes), respectively, were associated with NUE (Minic, 2008; Bi et al., 2009; Meijón et al., 2014; He et al., 2015; Jun et al., 2015; Marowa et al., 2016; Do et al., 2018; Zhang N. et al., 2020; Zhang Z. et al., 2020; Saini et al., 2021). Previous studies have also reported that genes governing glutamate synthase (GS) within QTLs interval are relevant for NUE and remobilization. These kinds of QTLs which are wider and have minor effects on N uptake should be verified by identification, isolation cloning, and genetic transformation.

As a result, breeding efforts should be more focused on regulatory proteins to get better results in terms of improved NUE. The majority of studies pertaining to the identification of markers or genes for NUE have been conducted at one level of N, however, missing out on information on physiological NUE. Another stumbling block to applying such results across an area and population is the substantial variability in N concentration of unfertilized soil. Even the validation of QTLs through gene cloning necessitates highly accurate phenotyping and genetic resources. The gene like NAC transcription factor in emmer and durum wheat has been identified as a marker for NUE which influence grain N content, zinc (Zn), and iron (Fe) concentration at the time of senescence by delaying the senescence period (Uauy et al., 2006). The better application of molecular markers for enhancing NUE genes in breeding methodologies and for transgenics requires quick, robust, and reliable phenotyping. Some genomic regions like GS and GOGAT controlling NUE are conserved across many crops like rice, maize, sorghum, and stiff broom grass; other than these conserved regions genes regulating photoperiod, vernalization, and semi-dwarf are also associated with NUE (Quraishi et al., 2011; Ranjan and Yadav, 2019). The N uptake system of plants uses two types of systems; one is a low-affinity transport system (LAST) and another a high-affinity transport system (HATS), these are more dominant when the plant faces N scarcity under limiting N conditions. *NRT1* gene families are responsible for coding LATS and *AtNRT1.1* and *AtNRT1.2* govern the nitrate uptake. The transcription factor *TaNCA2-5A* plays a significant role in the expression of transporters associated with nitrate uptake and happens to be a key controller in N supervisory system (He et al., 2015). The important QTLs related to NUE in cereal crops are highlighted in Table 3.

**TABLE 3:** Crucial root traits associated with improved nitrogen use efficiency.

Root traits	Crop	Gene associated	Role in improving NUE	References
Epidermis, phloem-companion cells, and xylem parenchyma	*Oryza sativa*	*OsNPF2.4*	Gene functions in the low acquisition affinity and long-distance transportation of NO^–3^	Xia et al., 2015
Lateral root primordia and vascular tissues of root	*Oryza sativa*	*OsATM1.3*	NH^+4^ transporter	Ferreira et al., 2015
Parenchyma cells of root	*Oryza sativa*	*OsNPF2.2*	Mutants of OsNPF2.2 were observed to maintain high nitrate level in roots than control plants	Li et al., 2015
Root sclerenchyma, stele, and cortex	*Oryza sativa*	*OsNPF7.2*	*OsNPF7.2* is a low affinity transporter of nitrates and aids in intracellular transportation of nitrates in the roots	Hu et al., 2016
Root epidermis	*Oryza sativa*	OsATM1.1	Dual-affinity transporter of NH^+4^ as well as root-to-shoot nitrate transporter	Li C. et al., 2016
Root hairs	*Arabidopsis thaliana*	*TGA1/TGA4 and NTR1.1*	Nitrate increase the density of root hairs which in turn increase the capacity of nitrate uptake	Canales et al., 2017
Root tissues	*Brassica napus*	*NRT2*	Out of 17 NRT2 genes almost all were expressed under nitrogen starvation to enhance the efficiency of nitrogen uptake.	Tong et al., 2020

### Transgenic Development to Enhance Nitrogen Use Efficiency

Through the transgenic approach defect removal in any cell is made possible by integrating a single foreign gene sequence in the host’s genome background via transformation method is only a quick and time-efficient technique over the conventional methods. Many insect-pest resistant, disease resistant, and genetically modified crops have been released for their cultivation in 28 countries across the world: the United States, Brazil, Canada, Argentina, China, and India among them (FAOSTAT, 2016). Researchers have focused on seven genes namely *GS1*, *GS2*, *GOGAT*, *AlaAT*, *GDHA*, *Nia1*, and *Nia2* to develop genetically engineered NUE cereals mainly in rice, corn, wheat, and rapeseed-mustard. The major studies report that the gene used for improved NUE was mainly plant-based except for some bacterial genes and tested in both model plant Arabidopsis as well as in other crop plants also. The most extensively plant-based gene used for NUE was the gene that is involved in GS biosynthesis which makes GS a major source for N movement from source to sink. It has also been evident that over-expression of this gene in cereal crops, improves the NUE, foliage, and grain yield, however, it has not been reported in dicots due to mismatch or poor promoter (Ranjan and Yadav, 2019). A previous study reported that post-flowering expression of a *TaNRT2.1* was very well associated with uptake of nitrate, the gene code for root nitrate transporter, and correlated with high protein and grain yield in wheat (Taulemesse et al., 2015; Xing et al., 2019). The gene *OsNRT1 is* expressed under low N conditions in rice (Huang et al., 2009). *AtNRT2.1* and *AtNRT2.1* well correlated with nitrate uptake and showed 75% high affinity under a scarcity of N content, suggesting that expression of these two genes related with better performance under low N conditions in *Arabidopsis* (Li et al., 2007). In maize expression of two genes, *ZmNRT2.1* and *ZmNRT2.2* through sequence homology approach accounts for increased resistance to low N concentration (Plett et al., 2010) while the overexpression of genes *Gln1-3* and *Gln1-4* through virus promoter, which codes for GS biosynthesis, results in the 30% increase in maize kernel yield (Martin et al., 2006). In the rice plant, *OsNRT1.1B* and *OsNRT2.3b* were identified as nitrate transporter genes. By using this promoter gene, when barley *AlaAT* gene was transferred in canola and rice, enhanced the NUE by 40 and 12% in canola and rice, respectively, recently many private companies are using *OsNRT2.3b* in various crops like maize, rice wheat, and soybean to accelerate grain yield through improved NUE (Good et al., 2007; Hu et al., 2015; Feng et al., 2017; Wang et al., 2018; Sisharmini et al., 2019; Shah et al., 2021a,b). The AlaAT approach is less understood, if this method is used with the proper understanding of promoter can evolve better results. As discussed above, very few bacterial genes were used for enhancing NUE, a bacterial gene from *E. Coli*, *GDHA*, expressed in tobacco via plant virus promoter improved the biomass by 10% under deficient N concentration (Mungur et al., 2005, 2006). The researchers have reported that overexpression of *TaNAC2-5A* aided grain yield and N movement in wheat plants and suggested that the gene can be utilized in wheat breeding programs to increase grain production through the development of high NUE varieties (He et al., 2015). In tomato plants it is observed that over-expression of *LeNRT2.3* leads to enhance nitrate uptake and N movement (Fu et al., 2015). Genetically engineered Arabidopsis showed improved growth and vigor through altered N and C metabolism under low N conditions via over-expression of Dof1 (DNA-binding with one finger) (Yanagisawa et al., 2004) and also when engineered with *OsATG8b* gene of the rice plant, which plays a key role in N mobilization in rice plant (Zhen et al., 2019). Similar, findings were observed by Kurai et al. (2011) in rice. Transgenic rice with modified *OsENOD93-1* resulted in improved NUE under both the conditions low as well as in optimal N conditions (Bi et al., 2009). Similarly, genetically engineered rice exhibited improved NUE via over-expression of Rubisco enzyme (ribulose 1, 5- biphosphate carboxylase- oxygenase), which plays a vital role in photosynthesis (Yoon et al., 2020). The important trans-genes and transcription factors used for enhancing NUE and their mechanism are highlighted in Table 4.

**TABLE 4:** Transgenes introduced and their role in improving nitrogen use efficiency.

Genes	Crop	Function	Role in improving NUE	References
*NRT1.7*	*Arabidopsis* Tobacco Rice	Loading excess nitrate stored and facilitates nitrate allocation to sink leaves	Enhancing source-to-sink nitrate remobilization	Chen et al., 2020
*GS 1*	Rice	Glutamine synthetase (cytosol)	Increase in total N and A.A	Cai et al., 2009
GS 2	Rice	Glutamine synthetase (Plastid)	Increased in photorespiration	Hoshida et al., 2000
*TaNRT2.1*	Rice	Uptake of nitrate at post-flowering	high protein and grain yield	Taulemesse et al., 2015
*OsNRT2.1*	Rice	N-uptake and mobilization	Expressed during low N condition	Chen et al., 2016
*ARE1*	Rice	N-uptake and mobilization	increased NUE, delayed senescence, increased biomass	Zhang J. et al., 2021
*GOGAT*	Tobacco	Glutamate synthetase	Increased in Biomass	Chichkova et al., 2001
*AlaAT*	Mustard Rice	Alanine amino transferase	Increased in biomass and grain yield	Good et al., 2007; Sisharmini et al., 2019
*GDHA*	Tobacco	Glutamate dehydrogenase	High water potential during drought, Increased in biomass and dry weight, Increased in ammonium assimilation.	Ameziane et al., 2000; Mungur et al., 2005, 2006
*Nia1*	Lettuce	Nitrate reductase	Nitrate content	Lillo et al., 2003
*Nia2*	Potato	Nitrate reductase	Reduced in nitrate level	Djennane et al., 2002
*AtNRT2.1* *AtNRT2.2*	*Arabidopsis*	Nitrate uptake	75% high affinity under scarcity of N content	Li et al., 2007
*OsATG8b*	*Arabidopsis*	N remobilization	Tolerance to nitrogen starvation	Zhen et al., 2019
*Gln1-3 and Gln 1-4*	Maize	Glutamate synthetase	30% more grain yield in Low N uptake	Martin et al., 2006
*ASN1*	Rice	Asparagine synthetase 1	Improve N content in grain	Lee et al., 2020
*DEP1*	Rice	DENSE AND ERECT PANICLES 1	Ammonium uptake and assimilation	Sun et al., 2014
*TaVRN1*	Wheat	VERNALIZATION1	Improve NUE	Lei et al., 2018
**Transcript factors**				
*ENOD93–1*	Rice	Early nodulin	Increased in biomass and seed yield	Bi et al., 2009
*TOND1*	Rice	Tolerance of Nitrogen deficiency	Increased the tolerance to N deficiency	Zhang Y. et al., 2015
*NAC*	Wheat	Influence grain N content, zinc (Zn) and iron (Fe) concentration	Delaying the senescence period	Uauy et al., 2006
*MADS25*	Rice	Promote lateral and primary root development and improve root attributes	Increase the expression of nitrate transporter genes	Yu et al., 2015

The increase in N uptake from the seedling stage to the pre-flowering stage and increased N-translocation at the post-flowering stage are the two ways to improve NUE in any plant. Because there is a link between N uptake and rhizosphere nitrification, as well as the contribution of nitrate to N uptake, current high yield-crop breeding initiatives that modify the expression of nitrate transporters in new crops will enhance the balance between nitrate-N and ammonium-N uptake. Anticipating, germplasm-related differences in nitrate transporter activity will be revealed in the future, and employing transporters to improve N absorption and translocation, and therefore increase NUE, will be discovered.

### Omics Approaches for Enhancing Nitrogen Use Efficiency

By virtue of the necessity of precise identification of genes involved in N uptake, mobilization, and recycling at various plant growth stages from seedling to maturity, taking the benefits of many omics data sets that include transcriptomics, proteomics, and metabolomics which could further be accessed in an interactive manner by using bioinformatics, computational, and mathematical techniques. Previous researchers have investigated rice transcriptome under N deficient conditions by using high scale data set to identify the specific N responsive genes and miRNA (Lian et al., 2006; Bi et al., 2009; Jeong et al., 2011; Cai et al., 2012; Yang et al., 2015).

However, these findings were restricted to single type RNA like, mRNA or small RNAs, and also have not provided an overview of transcriptome responses to alter N mobility. Shin et al. (2018) examined the transcriptomic analysis by using non-coding RNAs (lncRNAs) under low N conditions and the results provided the information on transcriptomic wide modification and response under N scarcity situation. In the future this large-scale transcriptomic data set will provide important information for the development of improved NUE- rice varieties.

Tiwari et al. (2018) integrated genomics with physiological and breeding approaches for improving NUE in potato. Whole genomic and transcriptomic approaches have identified the mRNA transcripts and TFs for NO_3_^–^ sensing and signaling in *Arabidopsis* (Marchive et al., 2013). Amiour et al. (2012) identified detailed mechanisms of N metabolism by integrating metabolomics transcriptomic and proteomic approaches in maize. The first investigation on complete gene-to-metabolite networks controlling NUE in *Arabidopsis* was reported by Hirai et al. (2004). Simons et al. (2014) developed a comprehensive understanding of N regulation and metabolism in maize by involving available transcriptome, proteome, and metabolome datasets. Ichihashi et al. (2020) analyzed a *Brassica rapa* based agroecosystem using multi-omics and integrated informatics approaches and revealed complex interactions among multiple network modules of NUE. Beatty et al. (2016) elucidated the whole-plant nitrogen metabolism using metabolomics and computational approaches in crops. Therefore, integrated omics is a holistic approach to understanding the N flow and associated regulation at the cellular, organ, and whole-plant levels.

## Conclusion and Future Prospects

Global food security with nutritional security is the most crucial task for future generations. However, the efforts of scientists or agricultural researchers can achieve this task and somehow they succeed in finding numerous technologies and ways through which they are trying to complete. Although, in modern times several new problems such as climate change, abiotic stresses, and problematic soils cause the loss of natural resources, and together amplify our food security target. In this scenario, there is an emergency need to reduce the cost of cultivation and degradation of natural resources. Therefore, increasing the NUE in economically important crops is a great challenge to secure environmental sustainability. However, the advancement in agronomical approaches has achieved some milestones in the last decades such as precision farming and nano-fertilizers. On the other hand, recent advancements in molecular biology and tools speed up the process and have a bright future in developing higher NUE efficient crops. However, still there is a huge gap in targeted NUE and also reduced during the last decades because of higher use of N fertilizers, especially urea. Therefore, it is recommended that the new fertilizer policy is based on the alternatives to urea for N sources such as customized, bio, and nano-fertilizers, which might be a breakthrough in enhancing NUE and reducing N pollution. Also, there might be a decision support system for best management practices at a local basis, which can directly benefit to the farmer in enhancing NUE and yield. Therefore, we combined these two crucial approaches in respect to improving NUE and might be game-changing for enhancing NUE in near future. Moreover, future trends and expectations should be aimed at cracking the current main hurdles to crop plants. Modern genome-editing tools can provide a permanent solution by developing varieties with enhanced NUE, although further studies are needed to reach these goals. The integration of all these approaches will lead to the sustainable production of crops, through the effective management of environmental stresses under the present scenario of changing climate. In addition, plant epigenetics, which is a conserved gene expression regulatory mechanism including histone modification, DNA methylation, non-coding RNA, and chromatin remodeling, represents an emerging and efficient tool to better understand biological processes in response to nitrogen enhancement.

## Author Contributions

TJ and RKS: conceptualization. II, TJ, RKS, RS, and AS: writing—original draft preparation. PK, AS, DJ, MAS, PD, DSa, HA, RA, and DSi: writing—review and editing. DSi and RA: funding acquisition. All authors have read and agreed to the published version of the manuscript.

## Conflict of Interest

The authors declare that the research was conducted in the absence of any commercial or financial relationships that could be construed as a potential conflict of interest.

## Publisher’s Note

All claims expressed in this article are solely those of the authors and do not necessarily represent those of their affiliated organizations, or those of the publisher, the editors and the reviewers. Any product that may be evaluated in this article, or claim that may be made by its manufacturer, is not guaranteed or endorsed by the publisher.
